# Supplementary material for: Giant electron–phonon coupling of the breathing plane oxygen phonons in the dynamic stripe phase of La1.67Sr0.33NiO4
Source: Sci Rep. 2020 Jul 10;10:11426. doi: 10.1038/s41598-020-67963-x (PMC7351770; doi:10.1038/s41598-020-67963-x)
Supplement: Supplementary file 1 — Supplementary information. [file 41598_2020_67963_MOESM1_ESM.pdf]

Giant electron-phonon coupling of the breathing plane oxygen phonons in the dynamic stripe phase of $La_{1.67}Sr_{0.33}NiO_4$

Supplementary Material

Temperature-dependence of the high energy LO phonons in $La_{1.7}Sr_{0.3}MnO_4$

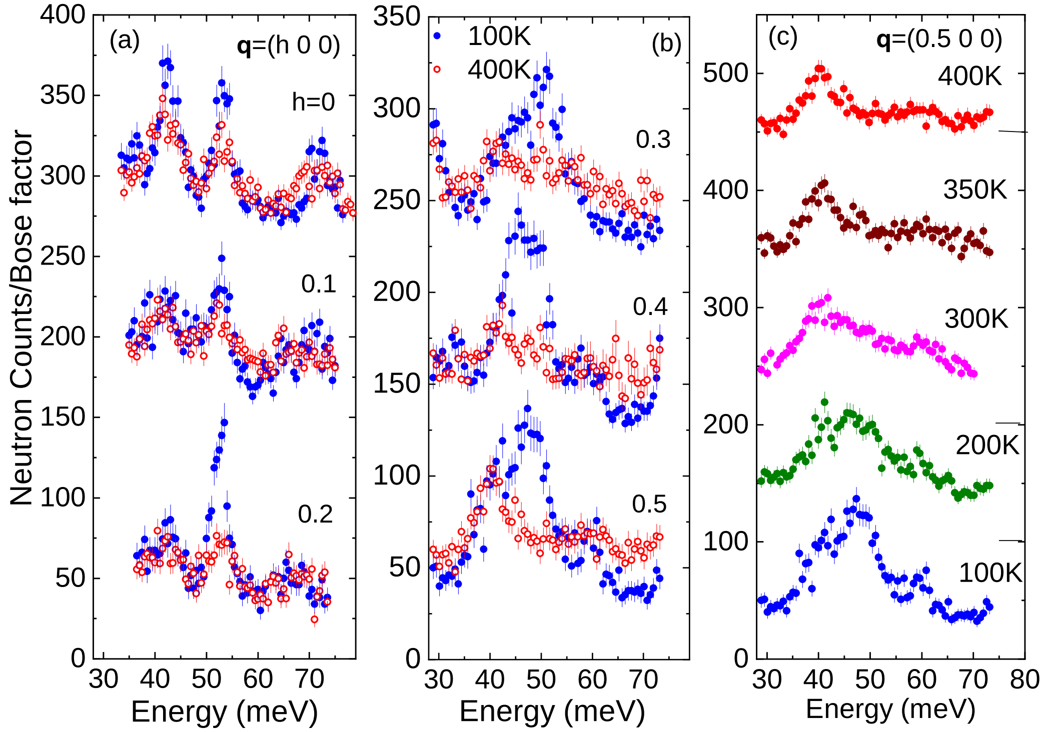

Figure S1: LO phonons in $La_{0.7}Sr_{0.3}MnO_3$. Data were obtained at **Q**=(4 0 0) +**q**. (a,b) LO phonon spectra as a function of wavevector at 100K and 400K. (c) Temperature-dependence of the zone-boundary phonons. Note how the bond-stretching (half-breathing) phonon at 49meV becomes overdamped above the FM transition temperature of 350K.

Interplay between the charge, spin, and lattice degrees of freedom in perovskite manganites results in a multitude of unconventional properties of fundamental as well as practical interest. Ferromagnetic alignment of Mn core spins appears at some stoichiometries due to the double exchange interaction and the ferromagnetic- paramagnetic (FM) transition in these systems is accompanied by large magnetoresistance (MR). Polarons forming and condensing above the (FM) transition temperature, T_C, have been observed by neutron scattering in $La_{0.7}Ca_{0.3}MnO_3$ and other manganites exhibiting Colossal Magnetoresistance (CMR) (Ref. 53 in main paper).

Here we show that half-breathing phonons in $La_{1-x}Sr_xMnO_3$ (x=0.3) become overdamped on

approach to the zone boundary much like the breathing phonons in LSNO. $La_{0.7}Sr_{0.3}MnO_3$ has a cubic perovskite structure except for a rotation of the MnO_6 octahedra around the 111 direction, which makes it rhombohedral. In addition, the crystals are twinned, so one always has to consider the effect of the superposition of the two domains. To simplify the analysis of our results we will use the cubic notation and treat the rotation of the octahedra as a rhombohedral distortion. Longitudinal branches in the (1 0 0) direction shown in Fig. S1 are the same for the two domains, so there are no complications due to twinning, which is why our measurements focused on this direction.

The experiment was carried out on the triple- axis spectrometer 1T located at the ORPHEE reactor using doubly focusing Cu111 monochromator crystals and PG002 analyzer fixed at 14.8 or 30.5 meV. Our sample was a high quality single crystal of $La_{0.7}Sr_{0.3}MnO_3$ with the FM transition temperatures measured at 355K. The volume of the crystal was $0.5cm^3$. The neutron scattering spectra were divided by the Bose factor in order to compare one-phonon scattering intensities at dif- ferent temperatures. Two pyrolytic graphite (PG) filters were installed in the scattered beam to avoid second and third order scattering by the analyzer.

Low temperature (100K) data in Fig. S1 are reproduced from Ref 1. To summarize the published low-temperature results, there are four branches in the investigated energy range, all of oxygen character. Two of them are the bond-stretching and bond-bending branches that exist in the cubic perovskite structure. The other two are folded in by the tilt of the MnO_6 octahedra responsible for the rhombohedral structure. At the zone center one of the folded-in modes has a vanishing structure factor, whereas the other has a vanishing structure factor at the zone boundary. At the zone center the phonons are at 42meV(bond bending), 53meV(folded in), and 72 meV(bond-stretching). The bond-bending branch disperses sharply upwards, the bond-stretching branch disperses downwards, whereas the folded in mode is approximately flat. They cross near the reduced wavevector **q**=(0.15,0,0).

Here we focus on the temperature-dependence of the phonons on heating from 100K, which corresponds to the ferromagnetic metallic phase, to 400K, where the material is paramagnetic and polaronic. Phonons at the zone center (h=0) broaden and soften, which is qualitatively consistent with increased anharmonicity (See Fig. S1a). However, the phonons that have the bond-stretching character away from the zone center, i.e. modes of breathing character, display a dramatic loss of intensity. Note that due to branch anticrossing, the mode at $h \geq 0.2$ near 50-55 meV has a large breathing character. [1] The intensity around 55meV is strongly reduced at 400K at larger h. (Fig. S1b) The behavior is more clear at the zone boundary (h=0.5) where there are only 3 phonons instead of four at $0<h<0.5$ [1]. Fig. S1c shows that the bond-stretching phonon at 49meV at 100K effectively disappears from the spectra at and above the Curie temperature of 350K as the polaronic phase develops. This phonon’s eigenvector is very close to the half-breathing phonon illustrated in Fig. 1b in the main paper. The effect here is much stronger than in the half-breathing phonon in LSNO, which broadens and softens substantially but does not disappear. It is similar though to the breathing phonon in LSNO (Fig. 1c in the main paper), which becomes overdamped.

Finally we note that the result on LCMO in Ref. 51 (main paper) seems to be different from LSMO (Fig. S1a) in that the zone center phonon seems to disappear above the Curie temperature in the former there but not in the latter. However, we found that the zone center data become contaminated by third-order scattering from the analyzer at high temperature on the high-energy side of the phonon above 75meV. It is necessary to use 2 PG filters to avoid this contamination as we have done. Only one filter was used in Ref. 51, which is insufficient to remove this contamination completely at high temperature. Therefore, it looks like the zone center peak disappears, but it does not.

Reference:

[1] D. Reznik and W. Reichardt, Phys. Rev. B **71**, 092301 (2005).
